# 2-Keto-L-Gulonic Acid Improved the Salt Stress Resistance of Non-heading Chinese Cabbage by Increasing L-Ascorbic Acid Accumulation

**DOI:** 10.3389/fpls.2021.697184

**Published:** 2021-11-04

**Authors:** Mingfu Gao, Hao Sun, Meijun Shi, Qiqi Wu, Dongxu Ji, Bing Wang, Lixin Zhang, Yang Liu, Litao Han, Xicheng Ruan, Hui Xu, Weichao Yang

**Affiliations:** ^1^Key Laboratory of Pollution Ecology and Environmental Engineering, Institute of Applied Ecology, Chinese Academy of Sciences, Shenyang, China; ^2^University of Chinese Academy of Sciences, Beijing, China; ^3^CAS Engineering Laboratory for Green Fertilizers, Institute of Applied Ecology, Chinese Academy of Sciences, Shenyang, China; ^4^State Key Laboratory of Bioreactor Engineering and School of Biotechnology, East China University of Science and Technology (ECUST), Shanghai, China; ^5^Yikang Environment Biotechnology Development Co., Ltd, Shenyang, China

**Keywords:** 2-keto-L-gulonic acid, L-ascorbic acid, organic acid, salt stress, lipid peroxidation, non-heading Chinese cabbage

## Abstract

Salt stress has long been a prominent obstacle that restricts crop growth, and increasing the L-ascorbic acid (ASA) content of crops is an effective means of alleviating this stress. 2-Keto-L-gulonic acid (2KGA) is a precursor used in industrial ASA production as well as an ASA degradation product in plants. However, to date, no study has investigated the effects of 2KGA on ASA metabolism and salt stress. Here, we evaluated the potential of using 2KGA to improve crop resistance to salt stress (100mM NaCl) through a cultivation experiment of non-heading Chinese cabbage (*Brassica campestris* ssp. *chinensis*). The results showed that the leaf and root biomass were significantly improved by 2KGA application. The levels of metabolites and enzymes related to stress resistance were increased, whereas the hydrogen peroxide (H_2_O_2_) and malondialdehyde (MDA) contents were decreased. Lipid peroxidation and cell membrane damage were alleviated following 2KGA treatment. Positive correlations were found between photosynthetic pigments and organic solutes, ASA and photosynthetic pigments, and ASA and antioxidant enzymes. In contrast, negative correlations were observed between antioxidant enzymes and H_2_O_2_/MDA. Moreover, the expression levels of *L-gulono-1,4-lactone oxidase*, *GDP-mannose pyrophosphorylase*, *dehydroascorbate reductase-3*, and *ascorbate peroxidase* were increased by 2KGA treatment. These results suggested that exogenous 2KGA application can relieve the inhibitory effect of salt stress on plant growth, and the promotion of ASA synthesis may represent a critical underlying mechanism. Our findings have significant implications for the future application of 2KGA or its fermentation residue in agriculture.

## Introduction

Abiotic stress refers to specific environmental factors that are unfavorable for plant survival and development, such as high temperature, drought, and saline-alkali soils (Zandalinas et al., 2020). There are large areas of saline-alkali soils globally, and crops grown in these soils usually have a low yield and are of poor quality (Zhao et al., 2014). Moreover, hydroponic agriculture with desalinated seawater is also associated with the detrimental effects of salt stress (Santos et al., 2019). These observations highlight that saline-alkali soils or environments constitute key factors that restrict the sustainable development of agriculture.

L-ascorbic acid (ASA, vitamin C) is a non-enzymatic antioxidant commonly found in plants and plays a vital role in protecting plants from oxidative damage caused by abiotic stress. ASA is employed as an electron donor by ascorbate peroxidase (APX; EC 1.11.1.11), which catalyzes the conversion of hydrogen peroxide (H_2_O_2_) to water and O_2_ (Tyagi et al., 2020). Simultaneously, humans must obtain ASA from the diet, especially vegetables and fruits, to maintain good health. Current evidence shows that the fluctuation of ASA metabolism in plants greatly influences the resistance of plants to salt stress. Several studies have emphasized that increasing ASA levels can strengthen the salt stress tolerance of plants. Liu et al. (2013) demonstrated that, in tobacco, the knock-in of cDNA encoding L-galactose-1,4-lactone dehydrogenase (GLDH; EC 1.3.2.3), which catalyzes the conversion of L-galactono-1,4-lactone to ASA, increased *GLDH* expression and ASA content, thereby enhancing salt stress tolerance in the plant. Similarly, the overexpression of *dehydroascorbate reductase* [encoding dehydroascorbate reductase (EC 1.8.5.1), the enzyme that catalyzes the regeneration of ASA from dehydroascorbic acid] in rice also increased ASA content, consequently enhancing the adaptability of rice to salt stress (Kim et al., 2014). Moreover, the application of exogenous ASA can also increase the ASA content and salt stress resistance of plants (Shalata and Neumann, 2001). Despite these benefits of ASA, concerns regarding the use of genetically modified crops and the inherent instability of ASA have complicated their application in agriculture. Additionally, low ASA synthesis in plants under abiotic stress can greatly inhibit their growth and development (Huang et al., 2005; Gao and Zhang, 2008). Combined, the above evidence suggests that increasing the level of ASA can enhance the ability of plants to resist salt stress.

2-Keto-L-gulonic acid (2KGA) is an intermediate metabolite of ASA metabolism in plants and is produced *via* the degradation of ASA (Jia et al., 2019). In grape tissues, 2KGA is used to synthesize tartaric acid (Jia et al., 2019). However, no study to date has investigated the effects of 2KGA on ASA metabolic activities or plant growth and development. Interestingly, 2KGA, mainly produced *via* two-step microbial fermentation using sorbitol as a substrate, is also a key precursor used in the chemical synthesis of ASA (Yang et al., 2017). Currently, the global production of 2KGA is approximately 160,000–180,000 tons per year (Xu et al., 2021), and the application of this organic acid is merely limited to the industrial production of ASA. In addition, more than 50,000 tons of fermentation residue are discarded every year from the ASA industry in China (Kong et al., 2014), with 2KGA accounting for approximately 25–30% of this residue. Unlike many industrial waste products, this fermentation residue does not contain harmful chemicals or an excess of heavy metals; nonetheless, it is now treated as a wastewater (Xu et al., 2021). One study showed that treatment with the 2KGA-containing fermentation residue can increase the biomass and ASA content of plants in saline soil (Kong et al., 2014), suggesting that it has potential for agricultural application. However, this fermentation residue is a solution comprising a mixture of organic acids, such as 2KGA, oxalic acid, formic acid, and acetic acid, among others. To assess its suitability for use in agriculture, it is necessary to investigate the effects of 2KGA in isolation.

Non-heading Chinese cabbage (*Brassica campestris* ssp. *chinensis*) is widely cultivated in China, including in hydroponic agriculture. In this study, this crop plant was used as the research material to determine whether 2KGA can enhance the resistance of crops to salt stress. In addition to ASA, organic solute [e.g., soluble carbohydrates (SC), soluble proteins (SP), and proline] and photosynthetic pigment contents and the activities of antioxidant enzymes [e.g., superoxide dismutase (EC 1.15.1.1), peroxidase (EC 1.11.1.1), APX, and catalase (EC 1.11.1.6)] are also known to be important for plant resistance to salt stress (Nounjan et al., 2012; Ahanger et al., 2019); here, these parameters were also examined to evaluate the potential of 2KGA in relieving salt stress in plants. Furthermore, we measured the expression levels of genes involved in the ASA biosynthesis and recycling pathways to reveal the effect of 2KGA treatment on plant ASA metabolism under salt stress. The aim of this study was to provide a basis for the future application of 2KGA or its fermentation residue in agriculture.

## Materials and Methods

### Plant Materials and Cultivation Conditions

**Figure 1:**
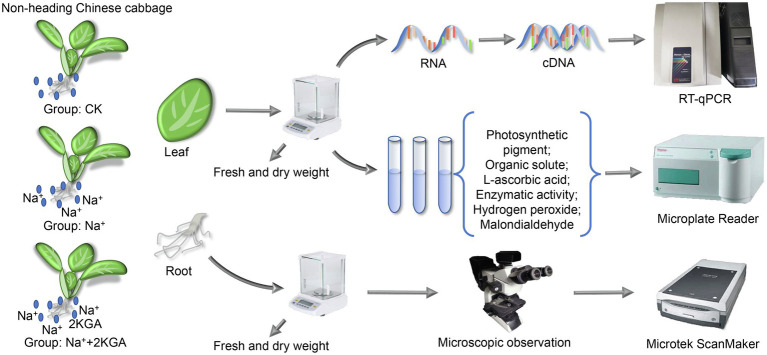
The experimental setup of the study. 2KGA, 2-keto-L-gulonic acid. CK, seedlings were cultivated in 1/2 Hoagland nutrition solution; Na^+^, the 1/2 Hoagland nutrient solution was supplemented with NaCl (100mM); and Na^+^+2KGA, 1/2 Hoagland nutrient solution was supplemented with NaCl (100mM) and 2KGA (1mM).

The experiment was conducted in a laboratory of the Institute of Applied Ecology, Chinese Academy of Sciences, Shenyang, China. 2KGA (>99.4%) was supplied by Northeast Pharmaceutical Group Co., Ltd., Shenyang, China. Non-heading Chinese cabbage seeds were soaked in 8% sodium hypochlorite solution for 10min, washed with desalinated water at least three times, and then placed on moist germination paper. After 6days, the cabbage seedlings at the first new leaf stage were transplanted into 1/2 Hoagland nutrient solution. When the third new leaf appeared, the seedlings were divided into the following three groups (90 seedlings per group): Group 1 (CK group), in which cabbage seedlings were transplanted into fresh 1/2 Hoagland nutrition solution as a control; group 2 (Na^+^ group), in which the fresh 1/2 Hoagland nutrient solution was supplemented with NaCl (final concentration: 100mM); and group 3 (Na^+^+2KGA group), in which the fresh 1/2 Hoagland nutrient solution was supplemented with NaCl (final concentration: 100mM) and 2KGA (final concentration: 1mM; Figure 1). The cultivation conditions of the seedlings were as previously reported (Lacerda et al., 2003). During the experiment, the temperature was set at 26±2°C, the relative humidity at 60±10%, and the illumination at 8,000lx. Plant samples were collected on days 3, 6, and 9 after the nutrient solution was replaced.

### Analysis of Photosynthetic Pigments

The assay for photosynthetic pigment content was based on the method of Lichtenthaler (1987). Photosynthetic pigments were extracted from leaves with 95% ethanol. The absorbance of the extract was measured at the 470, 649, and 665nm wavelengths. Full details are provided in Supplementary Method 1. Chlorophyll a (Chla), chlorophyll b (Chlb), total chlorophyll (Chlab), and carotenoid (Car) contents were calculated according to the following formulas:


$$C_{\text{Chla}}=13.36\times A_{665}-5.19\times A_{649}\;\left(\mathrm{\mu g}/\mathrm{ml}\right)$$

$$C_{\text{Chlb}}=27.43\times A_{649}-8.12\times A_{665}\;\left(\mathrm{\mu g}/\mathrm{ml}\right)$$

$$C_{\text{Chlab}}=5.24\times A_{665}+22.24\times A_{649}\;\left(\mathrm{\mu g}/\mathrm{ml}\right)$$

$$C_{\mathrm{car}}=\left(1,000\times A_{470}-2.13\times C_{\text{Chla}}-97.64\times C_{\text{Chlb}}\right)/209\;\left(\mathrm{\mu g}/\mathrm{ml}\right)$$

### Leaf and Root Sampling

Leaves and roots were collected on day 9 for the determination of the relevant parameters. The length and width of the leaves were measured. Total root length (the sum of detectable primary and lateral root lengths), root tip number, root average diameter, and surface area were measured using a Microtek ScanMaker i800 plus scanner, and the related parameters were collected *via* the instrument’s supporting software. Root morphology was observed under a microscope. Fresh plant samples were directly weighed to record fresh weight and then dried to constant weight at 75°C to record dry weight. Fresh roots were soaked in desalinated water, placed at 4°C in the dark for 24h, and then weighed to determine root turgor weight. Root relative water content was calculated according to the reported formula (Annunziata et al., 2017):


$$\text{Root relative water content}=\left(\text{root fresh weight}–\text{root}\;\mathrm{dry}\;\text{weight}\right)/\left(\text{root turgor weight}–\text{root}\;\mathrm{dry}\;\text{weight}\right)\times 100\%.$$

### Determination of Soluble Carbohydrate, Soluble Protein, Proline, and ASA Content in the Leaves

Soluble carbohydrate content in the leaves was determined using the phenol-sulfuric acid method (Nielsen, 2010). Leaves (0.5g) were chopped and placed in a centrifuge tube containing 5ml of desalinated water. The tube was placed in a boiling water bath for 30min and then cooled to room temperature. Next, the samples were centrifuged at 7,200×*g* for 10min at room temperature. The supernatant was transferred to a graduated test tube, and desalinated water was added to a total volume of 10ml, yielding the extract solution. A 1-ml aliquot of extract, 0.5ml of phenol solution (9% *w*/*v*), and 2.5ml of concentrated sulfuric acid (98%) were placed in a stoppered glass test tube, mixing, and placed in a boiling water bath for 15min. After cooling to room temperature, the absorbance was measured at 490nm. SC content was calculated using a glucose standard curve.

Soluble proteins concentration was assessed using the Bradford assay (Bradford, 1976). Proline was extracted from the leaves using sulfosalicylic acid (3% *w*/*v*) and quantified based on a previous report (Ghoulam et al., 2002). Details of the method are provided in Supplementary Methods 2, 3.

Ascorbic acid content was measured using a HPLC as previously reported (Cronje et al., 2012), with some modifications. The leaves (0.3g) were ground with liquid nitrogen and dissolved in 5ml of 1% (*w*/*v*) metaphosphate in an ice bath. The homogenate was centrifuged at 7,200×*g* for 10min at 4°C, and the resulting supernatant was diluted with metaphosphate and filtered using a 0.22-μm injection filter. A 20-μl aliquot of the filtered solution was injected into an AQ-C18 column for analysis. The detection conditions were as follows: mobile phase, 95% 20mM phosphate buffer, pH 2.8±0.1, and 5% acetonitrile; flow rate, 1ml/min; temperature, 40°C; detection wavelength, 243nm. The ASA content of the samples was determined according to a standard curve.

### Antioxidant Enzyme Activity

Leaf samples (0.2g) in 2ml of PBS (20mM, pH 7.2±0.1) supplemented with 0.1mM EDTA-Na, 0.5mM ASA, and polyvinylpyrrolidone (0.1% *w*/*v*) were evenly ground in an ice bath using a mortar and pestle. The homogenate was centrifuged at 7,200×*g* for 10min at 4°C, and the supernatant was collected as the crude enzyme solution. The activities of superoxide dismutase (SODa), ascorbate peroxidase (APXa), catalase (CATa), and peroxidase (PODa) were assayed as previously described (Azevedo Neto et al., 2006; Wang et al., 2014). More details of the experiment are provided in Supplementary Method 4.

### Measurement of H_2_O_2_ and Malondialdehyde Levels

The H_2_O_2_ content was determined using potassium iodide (KI; Velikova et al., 2000). Leaf samples (0.5g) were ground in an ice bath with 5ml of trichloroacetic acid (TCA, 0.1% *w*/*v*). The homogenate was centrifuged at 7,200×*g* for 10min at 4°C, and 0.5ml of the supernatant was mixed with 0.5ml of 0.1mol/l potassium phosphate buffer (pH 7.0±0.1) and 1ml of 1mol/L KI solution and kept at room temperature in the dark for 1h. Absorbance was then determined at 390nm. The H_2_O_2_ concentration was calculated using a H_2_O_2_ standard curve.

Malondialdehyde (MDA) content was determined using the thiobarbituric acid (TBA) method (Li and Yi, 2012). One gram of sample was mixed with 2ml of TCA (10% *w*/*v*) and a small amount of quartz sand and ground to obtain the homogenate. Subsequently, 8ml of TCA (10% *w*/*v*) was added to allow the even mixing of the homogenate. The homogenate was then centrifuged at 7,200×*g* for 10min at 4°C, and the supernatant was used as the sample extract solution. Then, 2ml of the extract solution was added to 2ml of TBA (0.6% *w*/*v*) solution and the mixture were placed in a boiling water bath for 15min to initiate the reaction. The mixture was subsequently rapidly cooled and centrifuged at 7,200×*g* for 3min at room temperature. The absorbance of the supernatant was measured at 450, 532, and 600nm. The MDA concentration was calculated using the following formula:


$$C_{\mathrm{MDA}}=6.45\times \left(A_{532}-A_{600}\right)-0.56\times A_{450}\;\left(\text{μmol}/L\right)$$

### The Expression of Genes Related to ASA Accumulation

The expression levels of *L-gulono-1,4-lactone oxidase* (*GLO*), *GDP-mannose pyrophosphorylase* (*GMP*), *Myo-inositol oxygenase* (*MIOX*), and *GLDH* in the ASA biosynthesis pathway and *monodehydroascorbate reductase* (*MDHAR*), *dehydroascorbate reductase-1* (*DHAR1*), *dehydroascorbate reductase-3* (*DHAR3*), and *APX* in the ASA recycling pathway were examined. Total RNA was extracted from leaves according to the instructions of the SteadyPure Universal RNA Extraction Kit (Code No. AG21017; Accurate Bio, Inc., Hunan, China). cDNA was prepared using Evo M-MLV RT Premix for qPCR (Code No. AG11706; Accurate Bio, Inc., Hunan, China). qPCR was performed using the SYBR Green Premix Pro Taq HS qPCR Kit (Code No. AG11701; Accurate Bio, Inc., Hunan, China). Details of the primers used are shown in Supplementary Table S1. Relative gene expression levels were calculated using the 2^-ΔΔCt^ method (Livak and Schmittgen, 2001).

### Statistical Analysis

Two-tailed Student’s *t* tests were used for statistical analysis. A value of *p*<0.05 was considered statistically significant. Each parameter was evaluated in at least three biological replicates. Spearman’s method was used to analyze putative correlations among the metabolites. Cytoscape 3.7.2 was used to plot the correlation networks.

## Results

### Leaves and Photosynthetic Pigments

**Figure 2:**
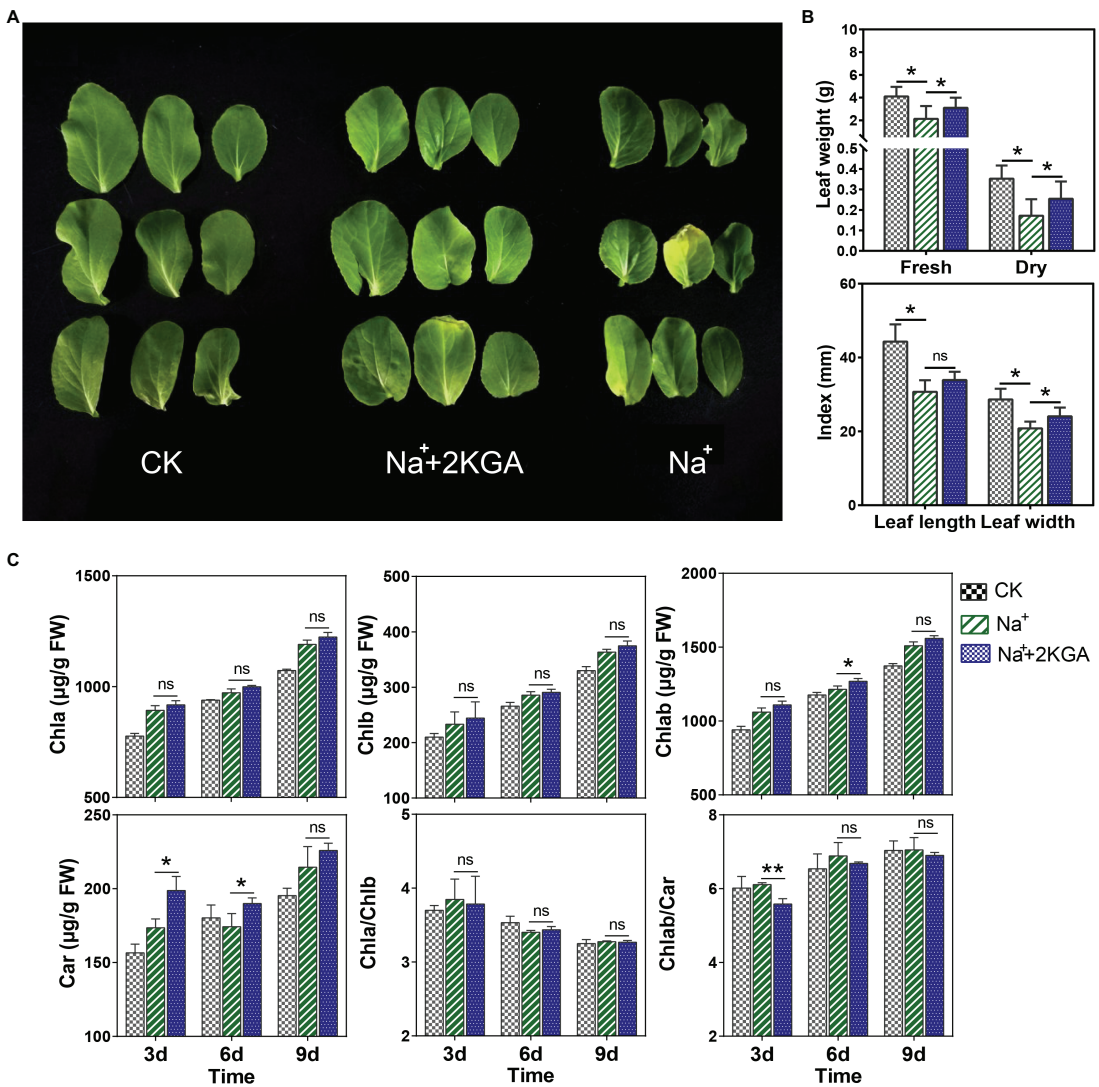
Leaf morphology, biomass, and photosynthetic pigment content. The morphology **(A)**, biomass, length, and width **(B)**, and photosynthetic pigment content **(C)** of the leaves. Chla, chlorophyll a; Chlb, chlorophyll b; Car, carotenoids; Chlab, total chlorophyll; Chla/Chlb, the ratio of chlorophyll a content to chlorophyll b content; Chlab/Car, the ratio of total chlorophyll content to carotenoid content; FW, fresh weight; and ns, not significant; ^*^p<0.05, ^**^p<0.01.

We found that salt stress significantly inhibited seedling growth. Compared with the CK (control) group, seedlings in the Na^+^ group were stunted and the leaves were significantly smaller (Figure 2A). Additionally, the fresh and dry weights of the salt-stressed leaves were reduced by 48.21 and 51.37%, respectively, compared with those of the CK group (Figure 2B). However, the addition of 2KGA (Na^+^+2KGA group) significantly increased the fresh weight and dry weight of the seedlings under stress by 45.77 and 48.44%, respectively, thereby effectively counteracting the negative impact of stress on seedling growth.

To improve the resistance of plants to salt stress, their photosynthetic efficiency must be enhanced by increasing the photosynthetic pigment content. Compared with those of the Na^+^ group, leaves in the Na^+^+2KGA group exhibited a greater accumulation of photosynthetic pigments. The Chlab content was also significantly greater in the leaves of the Na^+^+2KGA group on day 6; however, no difference in the Chla/Chlb ratio was observed between the two groups. Interestingly, 2KGA treatment led to a marked increase in carotenoid content, with the highest increase (12.5%) being recorded on day 3. Correspondingly, the Chlab/Car ratio of the Na^+^+2KGA group was lower (Figure 2C).

### Root Growth and Development

**Figure 3:**
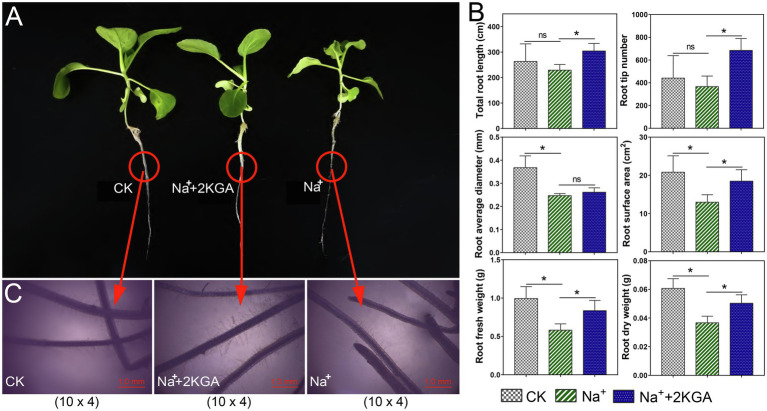
Root morphology and indexes for each experimental group. The morphology of intact plants **(A)** and roots (10×4; magnification: ×40; **C**), and root-related parameters **(B)**. Five root samples of each biological replicate were scanned. Total root length was calculated as the sum of detectable primary and lateral root lengths. ns, not significant, ^*^p<0.05.

Compared with the CK group, Na^+^-only treatment significantly reduced the root fresh weight and dry weight by 41.17 and 39.56%, respectively (Figures 3A,B), while the total root length, root surface area, average diameter, and root tip number were reduced by 12.89, 37.73, 32.90, and 16.94%, respectively. However, 2KGA supplementation relieved the inhibitory influence of salt stress on the roots. The corresponding root indices in the Na^+^+2KGA group showed increases of 43.73, 37.27, 32.72, 42.56, 5.92, and 86.86% compared to the Na^+^ group. Surprisingly, even under salt stress, the addition of 2KGA (Na^+^+2KGA group) increased the total root length and root tip number by 15.62 and 55.21%, respectively, compared with that of the CK group. Moreover, microscopic analysis showed that the roots of the Na^+^+2KGA group contained more and longer fine roots (Figure 3C). Unlike with the plant biomass, no significant changes in water content or root relative water content were recorded between the Na^+^ and Na^+^+2KGA groups (Supplementary Figure S1).

### Organic Solutes, ASA, Antioxidant Enzymes, H_2_O_2_, and MDA

**Figure 4:**
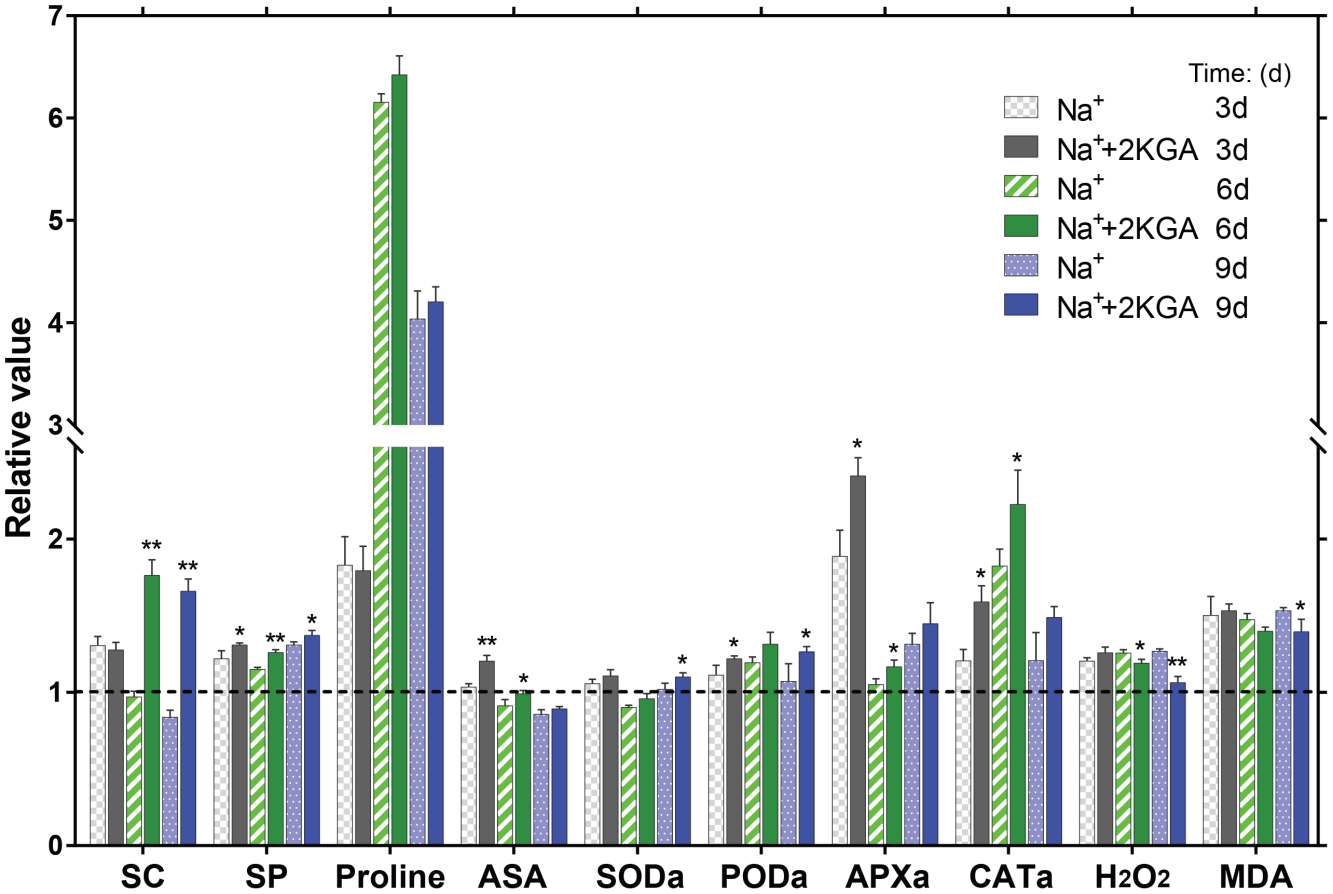
Variations in the contents of organic solutes, ASA, antioxidant enzymes, H_2_O_2_, and MDA in seedling leaves at different time points. Data were normalized relative to the control (CK) group. The absolute results are shown in Supplementary Table S2. SC, soluble carbohydrate; SP, soluble protein; ASA, L-ascorbic acid; SODa, superoxide dismutase activity; PODa, peroxidase activity; APXa, ascorbate peroxidase activity; CATa, catalase activity; H_2_O_2_, hydrogen peroxide; and MDA, malondialdehyde. ^*^p<0.05, ^**^p<0.01.

Under salt stress, plants must increase their levels of anti-osmotic organic solutes (such as SC, SP, and proline) to maintain cell morphology and balance osmotic pressure. In the early stage of stress (day 3), the SC concentration of the Na^+^ group increased by 30.47% compared with that of the CK group. Subsequently, however, the SC levels in the Na^+^ group decreased rapidly and were lower than those of the CK group on days 6 and 9. There was no significant difference in SC content between the Na^+^+2KGA and Na^+^ groups on day 3; however, the SC content in the Na^+^+2KGA group remained at a high level on days 6 and 9, showing increases of 81.68 and 98.03%, respectively, relative to those of the Na^+^ group (Figure 4).

The SP concentration displayed a continuous increase over time. The Na^+^+2KGA group had a greater SP content than the Na^+^ group, showing increases of 7.37, 9.53, and 4.74% on days 3, 6, and 9, respectively. From day 6, proline accumulation was significantly improved in the Na^+^ group and was 6.15-fold higher compared with that of the CK group. The proline content in the Na^+^+2KGA group was similar, albeit slightly greater, to that of the Na^+^ group. The ASA content of all the groups peaked on day 3. Compared with the Na^+^ group, the ASA content in the Na^+^+2KGA group was significantly increased (16.24%). After day 3, the ASA content of each group began to decline and was lower in the Na^+^ and Na^+^+2KGA groups than in the CK group on days 6 and 9; however, the ASA level was always higher in the Na^+^+2KGA group than in the Na^+^ group (Figure 4).

Superoxide dismutase was 7.90% higher in the Na^+^+2KGA group compared with that of the Na^+^ group on day 9. Meanwhile, PODa in the Na^+^+2KGA group was 9.63, 10.17, and 18.15% higher on days 3, 6, and 9, respectively, compared with that of the Na^+^ group. Additionally, compared with the Na^+^ group, APXa in the Na^+^+2KGA group was increased by 27.80, 11.03, and 10.29% on days 3, 6, and 9, respectively. Similarly, 2KGA caused a continuous increase in CATa of 31.85, 21.97, and 23.19% on days 3, 6, and 9 compared to that of Na^+^ (Figure 4).

In plants, MDA is a product of cell membrane lipid oxidation and is a biochemical marker for assessing the degree of membrane lipid peroxidation (Silva et al., 2010). The results showed that H_2_O_2_ and MDA remained at a stable level in the seedlings under normal growth conditions but increased under salt stress. In the early stage (day 3), 2KGA did not affect H_2_O_2_ metabolism; from day 6, however, the H_2_O_2_ level in the Na^+^+2KGA group decreased rapidly compared with that of the Na^+^ group and was reduced by 16.16% on day 9. Simultaneously, the MDA content decreased by 8.97% on day 9. These results indicated that membrane lipid peroxidation was attenuated following 2KGA treatment. The level of H_2_O_2_ in the Na^+^+2KGA group was similar to that of the control group on day 9 (Figure 4).

### Analysis of Correlations Among Leaf Metabolites

**Figure 5:**
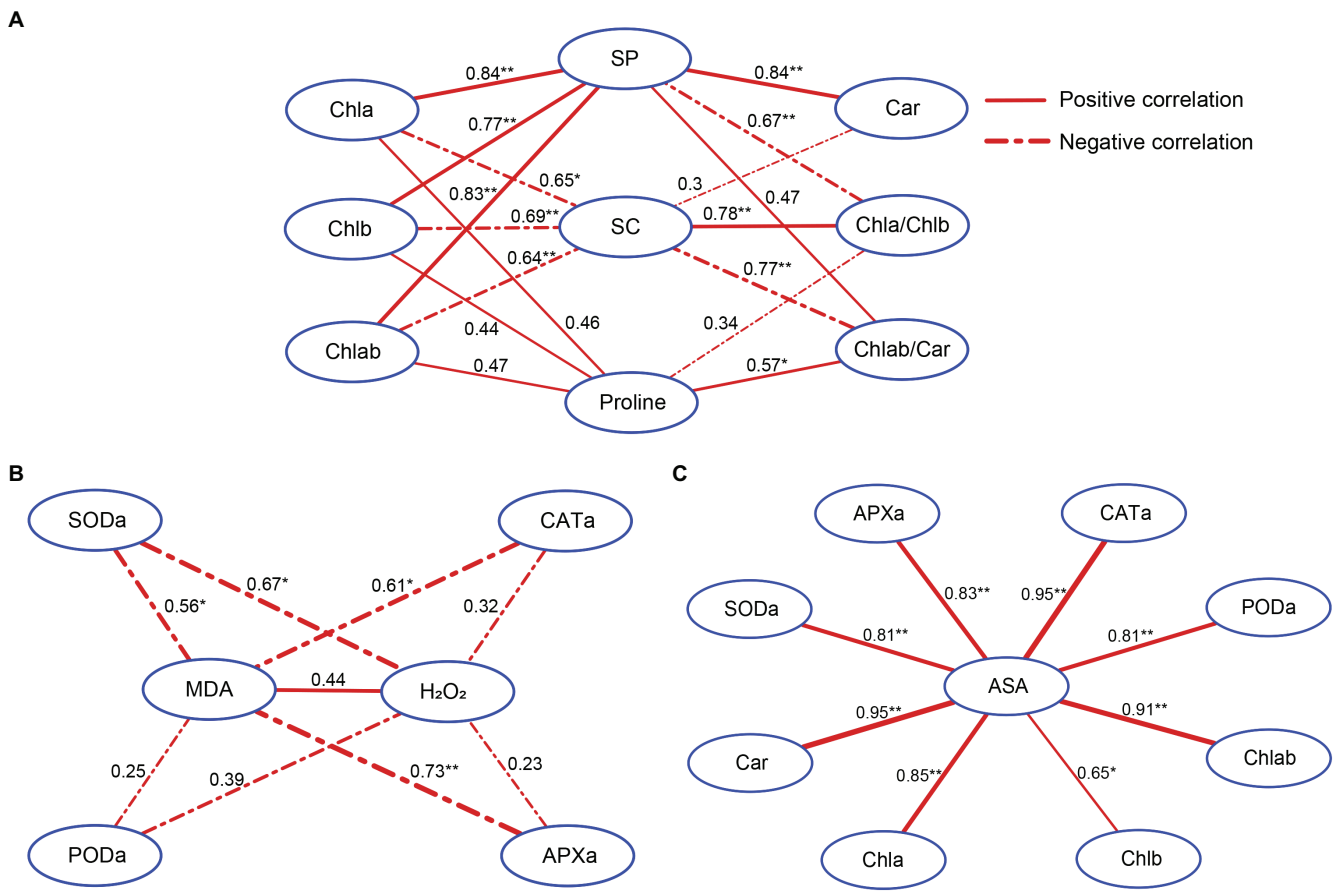
The correlations among different metabolites in seedling leaves. **(A)** The correlation between photosynthetic pigment and organic solute contents. **(B)** The correlation between antioxidant enzyme activities and H_2_O_2_/MDA levels. **(C)** The correlation among ASA, antioxidant enzyme activity, and photosynthetic pigment content. Chla, chlorophyll a; Chlb, chlorophyll b; Car, carotenoids; Chlab, total chlorophyll; Chla/Chlb, the ratio of chlorophyll a content to chlorophyll b content; Chlab/Car, the ratio of total chlorophyll content to carotenoid content; SC, soluble carbohydrate; SP, soluble protein; SODa, superoxide dismutase activity; PODa, peroxidase activity; APXa, ascorbate peroxidase activity; CATa, catalase activity; H_2_O_2_, hydrogen peroxide; MDA, malondialdehyde; and ASA, L-ascorbic acid. The data correspond to the absolute value of the correlation coefficient. ^*^p<0.05, ^**^p<0.01.

Under salt stress, variations in the levels of organic solutes and photosynthetic pigments and activities of antioxidant enzymes in leaves were correlated with H_2_O_2_ and MDA contents (Supplementary Figure S2). Under 2KGA treatment, changes in photosynthetic pigment levels were correlated with the osmotic resistance of organic solute contents (Figure 5A). The Chla/Chlb ratio was negatively correlated with SP and proline levels and positively correlated with SC levels; however, Chlab/Car levels were positively correlated with SP and proline content and negatively correlated with SC content. Antioxidant enzyme activity was negatively correlated with H_2_O_2_ and MDA concentrations (Figure 5B). ASA content was positively correlated with antioxidant enzyme activity and photosynthetic pigment levels (Figure 5C).

### ASA-Related Gene Expression

**Figure 6:**
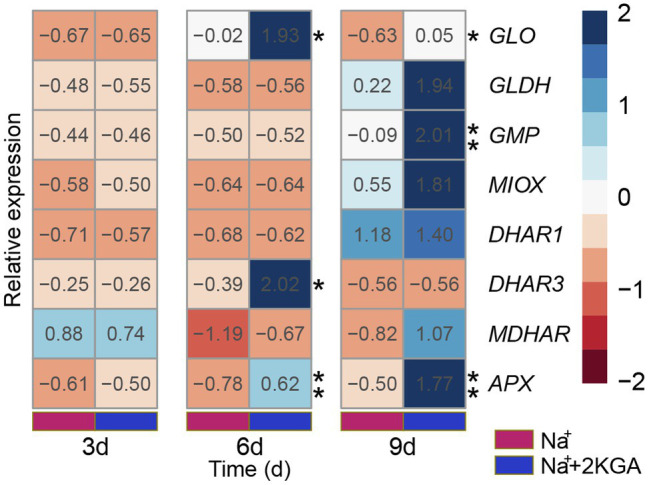
Relative gene expression related to ASA accumulation. Data were normalized relative to that of the control (CK) group. *GLO*, *L-gulono-1,4-lactone oxidase*; *GLDH*, *L-galactose-1,4-lactone dehydrogenase*; *GMP*, *GDP-mannose pyrophosphorylase*; *MIOX*, *Myo-inositol oxygenase*; *DHAR1*, *dehydroascorbate reductase-1*; *DHAR3*, *dehydroascorbate reductase-3*; *MDHAR*, *monodehydroascorbate reductase*; *APX*, *ascorbate peroxidase*. ^*^p<0.05, ^**^p<0.01.

The expression levels of *GLO* and *GMP*, genes acting in the ASA biosynthesis pathway, were significantly increased in the Na^+^+2KGA group relative to those of the Na^+^ group; in particular, the *GLO* expression level was consistently higher in the Na^+^+2KGA group than in the Na^+^ group (Figure 6). *MIOX* and *GLDH* levels were not significantly different between the Na^+^ and Na^+^+2KGA groups.

In the ASA recycling pathway, 2KGA treatment did not affect the expression of *MDHAR* or *DHAR1*. Compared to the Na^+^ group, the expression level of *DHAR3* was significantly increased on day 6 in the Na^+^+2KGA group, but no significant difference was observed between the two groups on day 9. Furthermore, the expression of *DHAR3* was lower in both the Na^+^ and Na^+^+2KGA groups than in the control group (*p*<0.05; Supplementary Figure S3). The expression of *APX* in the Na^+^+2KGA group was higher than that in the Na^+^ group at each sampling time point (Figure 6). The results of nucleic acid quality assessment are provided in Supplementary Table S3 and Supplementary Figure S4.

## Discussion

We have previously shown that a 2KGA-rich fermentation residue from the vitamin C industry could increase soil organic matter content and endogenous ASA concentrations in crops, thereby leading to increased crop yields in saline soil (Kong et al., 2014). However, this fermentation residue was a mixture, and its main effectors had not been identified. The 2KGA content in the residue varies between 25 and 30%, that of oxalic acid is approximately 1–2%, while the levels of other organic acids comprise less than 1%; much of the rest is water. This suggested that the 2KGA component of the residue might be a key to enhancing ASA production in plants. In this study, we found that 2KGA treatment increased the biomass of non-heading Chinese cabbage subjected to salt stress.

In this study, under salt stress, the levels of H_2_O_2_ and MDA in non-heading Chinese cabbage seedlings were significantly increased, and peroxidative damage in the cell membrane was aggravated, resulting in the inhibition of seedling leaf and root development. However, the observed increase in leaf and root biomass, especially that of roots, in the Na^+^+2KGA group suggested that 2KGA could effectively relieve this inhibitory effect and promote seedling growth and development. Because it has been shown that enhancing endogenous ASA levels can promote root development (Aghaei et al., 2008), we speculated that these effects were most likely related to an increase in ASA levels in the seedlings. In general, fluctuations in the concentrations of anti-osmotic organic solutes (including ASA), photosynthetic pigments, and antioxidant enzymes are essential for plants to resist salt stress.

Salt stress reduces the osmotic potential of plant cells by creating a high-salinity environment, which results in osmotic stress. The latter will lead to a reduction in the relative water content of plants and is not conducive to the maintenance of cell morphology (Annunziata et al., 2017). Osmotic adjustment represents an adaptive mechanism that helps cells maintain osmotic pressure and ensures normal metabolism and crop growth. In this study, we found that 2KGA treatment increased the SC, SP, and free proline contents in the leaves of non-heading Chinese cabbage under salt stress. The accumulation of SC is especially important for osmotic regulation, while SP and proline are believed to play a more important role in protecting cells from oxidative damage and enhancing defensive responses (Zhang et al., 2011; Li et al., 2019; Alfosea-Simón et al., 2020).

In the present study, the photosynthetic pigment content was found to be correlated with the SC level. When 2KGA was added under conditions of salt stress, the Chlab/Car ratio was negatively correlated with SC content. The results also showed that SC content was significantly increased on day 3 post stress induction and then rapidly decreased in the CK and Na^+^ groups, while the addition of 2KGA could effectively reverse the loss of SC content. Simultaneously, the Na^+^+2KGA group had a lower ratio of Chab/Car. Under all treatments, the photosynthetic pigment content continuously increased along with the growth of the seedlings. Under salt stress, photosynthetic pigments accumulated at a high level, and the variations in H_2_O_2_ and MDA contents were positively correlated with photosynthetic pigment levels. This indicated that enhanced photosynthesis was a defense mechanism employed by seedlings to resist salt stress, and the addition of 2KGA further promoted the accumulation of photosynthetic pigments. This was in agreement with the results of previous studies, in which an increase in photosynthetic pigment levels was shown to improve photosynthetic efficiency and promote plant growth (Garg et al., 2002; Li and Yi, 2020). In contrast, in a recent study, Gautam et al. (2020) found that the photosynthetic efficiency of tobacco was reduced under salt stress. Differences in crop species investigated, time of action, or salt stress intensity might explain these discrepant results.

Photosynthesis is the primary means by which plants obtain carbon sources in the environment. However, in this study, the increase in photosynthetic pigment concentrations did not improve SC content in seedlings. Interestingly, photosynthetic pigment levels were positively correlated with variations in SP and proline content, indicating that seedlings could respond to salt stress by increasing photosynthetic pigment production and adjusting the Chla/Chlb and Chlab/Car ratios. Combined, these observations suggested that the addition of 2KGA enhanced the ability of the seedlings to regulate photosynthetic pigment levels, thereby resisting salt stress.

The photosynthesis system is also one of the main sites of reactive oxygen species (ROS) production in plant cells (Foyer and Noctor, 2011). Here, we found that salt stress increased the H_2_O_2_ content. The accumulation of ROS leads to an increase in antioxidant enzyme activity (Attia et al., 2020; Li and Yi, 2020). Accordingly, PODa, CATa, and APXa were higher in the Na^+^ group than in the CK group and were positively correlated with fluctuations in H_2_O_2_ and MDA levels (Supplementary Figure S2). However, with the application of 2KGA, the activities of all four enzymes increased relative to those in the Na^+^ group, and they were all negatively correlated with H_2_O_2_ and MDA contents. These results suggested that salt stress promoted the increase in H_2_O_2_ and MDA concentrations in seedlings, which subsequently led to a passive increase in antioxidant enzyme activities. Nevertheless, the addition of 2KGA enhanced antioxidant enzyme activity in the leaves, leading to faster H_2_O_2_ and MDA clearance.

Our findings demonstrated that the application of 2KGA could significantly increase ASA concentrations in non-heading Chinese cabbage seedlings exposed to salt stress. ASA content was positively correlated with antioxidant enzyme activities and photosynthetic pigment levels, indicating that the increase in ASA levels promoted the increase in antioxidant enzyme activities and photosynthetic pigment contents in the seedlings. Plants use ASA to remove large amounts of ROS produced by the photosynthesis system to avoid peroxidative damage (Talla et al., 2011), which is indicative of the importance of ASA in the protection of the photosynthesis system. Lim et al. (2012) reported that under salt stress, increasing endogenous ASA content in the tomato could enhance the photosynthetic pigment content. Meanwhile, exogenous ASA application can reportedly increase the levels of endogenous ASA and the activities of antioxidant enzymes in plants under heavy metal stress and salt stress (Athar et al., 2008; Chao and Kao, 2010). The above results suggested that ASA has a crucial function in plant defenses against salt stress.

To explore the mechanism underlying the 2KGA-mediated enhancement of ASA synthesis in non-heading Chinese cabbage under salt stress, we analyzed the expression levels of eight genes involved in the ASA accumulation. *DHAR3* was more highly expressed in the Na^+^+2KGA group relative to that in the Na^+^ group, but only in the early stage of the experiment; in later stages, *DHAR3* expression was downregulated in both groups. This observation may explain why the ASA content was lower in the salt-stressed groups than in the CK group. The increase in *GMP* expression in the Na^+^+2KGA group reached significance only on day 9, and its contribution to ASA accumulation in the early stage was thus likely to have been limited. At present, GLO is the only confirmed enzyme involved in the synthesis of ASA in the L-gulose pathway. The expression level of *GLO* in the Na^+^+2KGA group was maintained at a higher level throughout the whole test period compared with that in the Na^+^ group, while greater ASA accumulation was also observed in the former. An earlier study on the potato showed that an increase in *GLO* expression promoted the accumulation of ASA and enhanced abiotic stress tolerance (Lim et al., 2012). As a downstream product of ASA metabolism, the ketone group of 2KGA can be reduced to a hydroxyl group (Jia et al., 2019), which has the possibility of forming gulonic acid, a precursor in gulonolactone synthesis (catalyzed by GLO and converted to ASA) in plants (Cruz-Rus et al., 2012). This suggests strongly that exogenous 2KGA supplementation may increase the endogenous 2KGA content in non-heading Chinese cabbage. Meanwhile, the higher levels of gulonic acid and gulonolactone, both ASA precursors, finally leads to an increase in ASA content *via* GLO catalytic activity. The increase in *GMP* and *DHAR3* expression levels also exerted a positive effect on plant ASA accumulation (Lin et al., 2011; Wang et al., 2019). Moreover, in agreement with the observed increase in APX enzyme activity, *APX* gene expression was found to be upregulated with 2KGA administration, which further implied that 2KGA enhanced the resistance of non-heading Chinese cabbage seedlings to salt stress by increasing the endogenous ASA content. APX catalyzes the conversion of H_2_O_2_ to water and O_2_, with ASA serving as the reductant (Tyagi et al., 2020). Thus, the higher ASA content and APXa in the Na^+^+2KGA group explains why the level of H_2_O_2_ was lower in this group than in the Na^+^ group.

**Figure 7:**
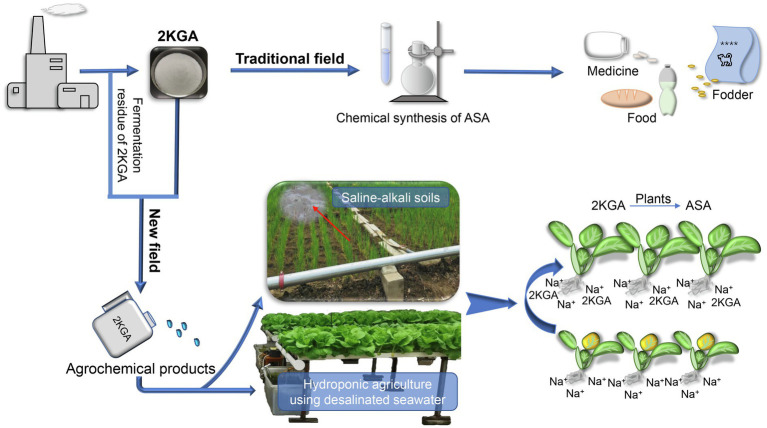
The prospect for the application of 2KGA and its fermentation residue. 2KGA, 2-keto-L-gulonic acid; ASA, L-ascorbic acid.

Although we demonstrated that 2KGA can relieve the inhibition of salt stress on the growth of non-heading Chinese cabbage seedlings, this is a preliminary study in this field. Many aspects remain to be explored, such as whether 2KGA has the same effect on different crops, whether 2KGA influences acetylsalicylic acid synthesis and carbon and nitrogen metabolism, and how 2KGA can efficiently be applied in agricultural practice. As a precursor of industrial ASA synthesis, 2KGA is produced on a large scale using a two-step microbial fermentation process; however, that 2KGA is only used in the chemical synthesis step of ASA production. In addition, a large amount of fermentation residue is discarded from the ASA industry. This provides a reliable industrial base for the application of 2KGA or its fermentation residue in a new field, that is, agriculture. Consequently, given the function of 2KGA against salt stress and its availability in the ASA industry, the prospect of applying 2KGA in agriculture, such as for crop cultivation in saline-alkali soils and hydroponic agriculture, merits further investigation (Figure 7).

## Conclusion

In summary, to the best of our knowledge, this is the first study to report that 2KGA relieves the inhibitory effect of salt stress on non-heading Chinese cabbage, which has laid the foundation for the future application of 2KGA in agriculture. Meanwhile, a potential novel direction for the study of plant ASA metabolism was also identified. Our findings suggest that exogenous 2KGA application strengthens non-heading Chinese cabbage defenses against salt stress, for which the promotion of ASA accumulation may represent a crucial underlying mechanism.

## Data Availability Statement

The original contributions presented in the study are included in the article/Supplementary Material; further inquiries can be directed to the corresponding authors.

## Author Contributions

MG and HX: conceptualization. MG, MS, QW, and DJ: methodology. HS: software. MG and MS: validation. MG and BW: formal analysis. YL, LH, and XR: resources. MG: investigation, data curation, writing-original draft, and visualization. MG, HX, HS, and WY: writing-review and editing. WY and LZ: supervision. HX: project administration. All authors contributed to the article and approved the submitted version.

## Funding

This work was supported by the National Key Research and Development Program of China (grant number 2020YFA0907800), the Science and Technology Plan Project of Shenyang (grant number 20-203-5-48), and the Open Research Project of Shouguang Facilities Agriculture Center in the Institute of Applied Ecology (grant number 2018SG-S-02).

## Conflict of Interest

YL, LH, and XR were employed by the company Yikang Environment Biotechnology Development Co., Ltd., Shenyang, China.

The remaining authors declare that the research was conducted in the absence of any commercial or financial relationships that could be construed as a potential conflict of interest.

## Publisher’s Note

All claims expressed in this article are solely those of the authors and do not necessarily represent those of their affiliated organizations, or those of the publisher, the editors and the reviewers. Any product that may be evaluated in this article, or claim that may be made by its manufacturer, is not guaranteed or endorsed by the publisher.
