# Supplementary material for: 2-Keto-L-Gulonic Acid Improved the Salt Stress Resistance of Non-heading Chinese Cabbage by Increasing L-Ascorbic Acid Accumulation
Source: Front Plant Sci. 2021 Nov 4;12:697184. doi: 10.3389/fpls.2021.697184 (PMC8599927; doi:10.3389/fpls.2021.697184)
Supplement: Supplementary file 1 [file Data_Sheet_1.PDF]

# *Supplementary Material*

## Supplementary Figures

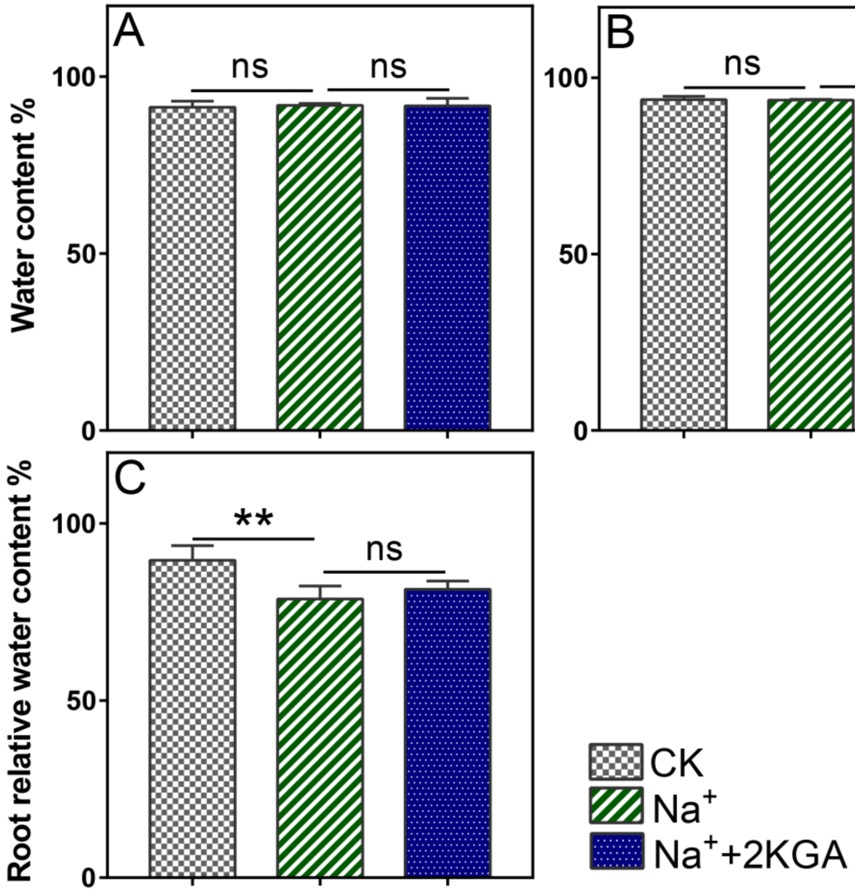


**Supplementary Figure 1.** Water content of leaf **(A)** and root **(B)**, and root relative water content **(C)**. Water content = (fresh weight – dry weight) / fresh weight × 100%. Root relative water content = (root fresh weight – root dry weight) / (root turgor weight – root dry weight) × 100%. CK, seedlings were cultivated in 1/2 Hoagland nutrition solution; $Na^+$, 1/2 Hoagland nutrient solution was supplemented with NaCl (100 mM); $Na^+$+2KGA, 1/2 Hoagland nutrient solution was supplemented with NaCl (100 mM) and 2KGA (1 mM). ns, not significant, ** $p < 0.01$.

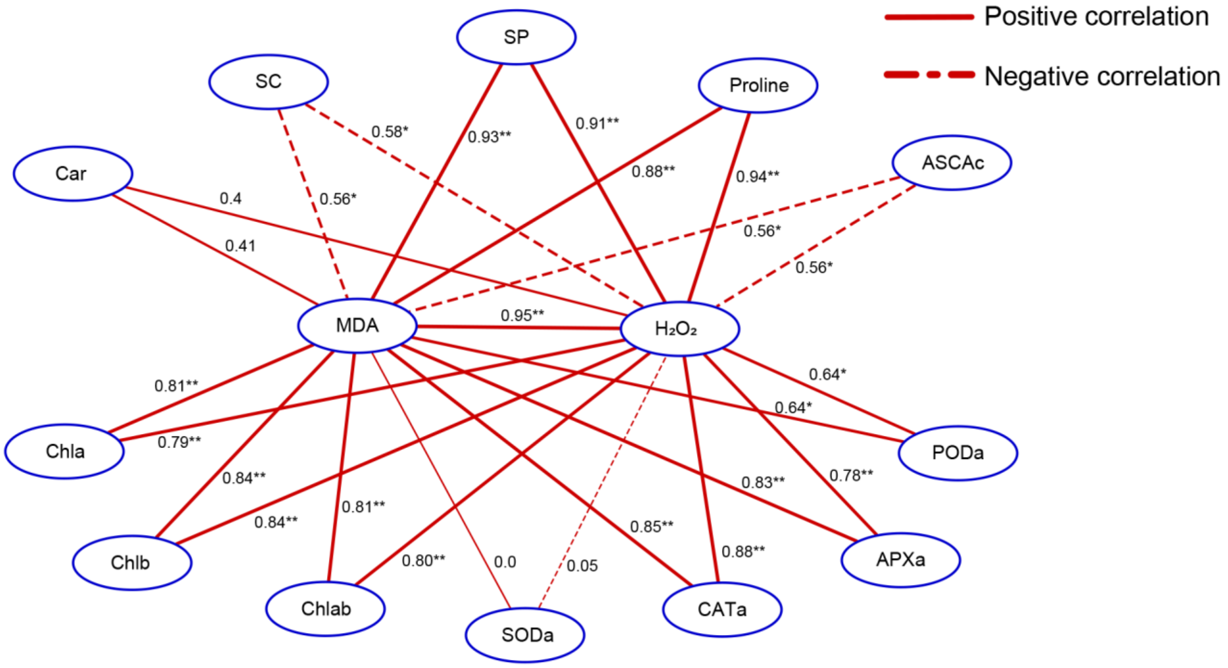


**Supplementary Figure 2.** Correlation of metabolites under salt stress without 2KGA. Chla, chlorophyll a; Chlb, chlorophyll b; Car, carotenoid; Chlab, total chlorophyll; Chla/Chlb, the ratio of chlorophyll a content to chlorophyll b content; Chlab/Car, the ratio of total chlorophyll content to carotenoids content; SC, soluble carbohydrate; SP, soluble protein; SODa, superoxide dismutase activity; PODa, peroxidase activity; APXa, ascorbate peroxidase activity; CATa, catalase activity; $H_2O_2$, hydrogen peroxide; MDA, malondialdehyde; ASCAc, L-ascorbic acid. The data corresponds to the absolute value of the correlation coefficient. * $p < 0.05$, ** $p < 0.01$.

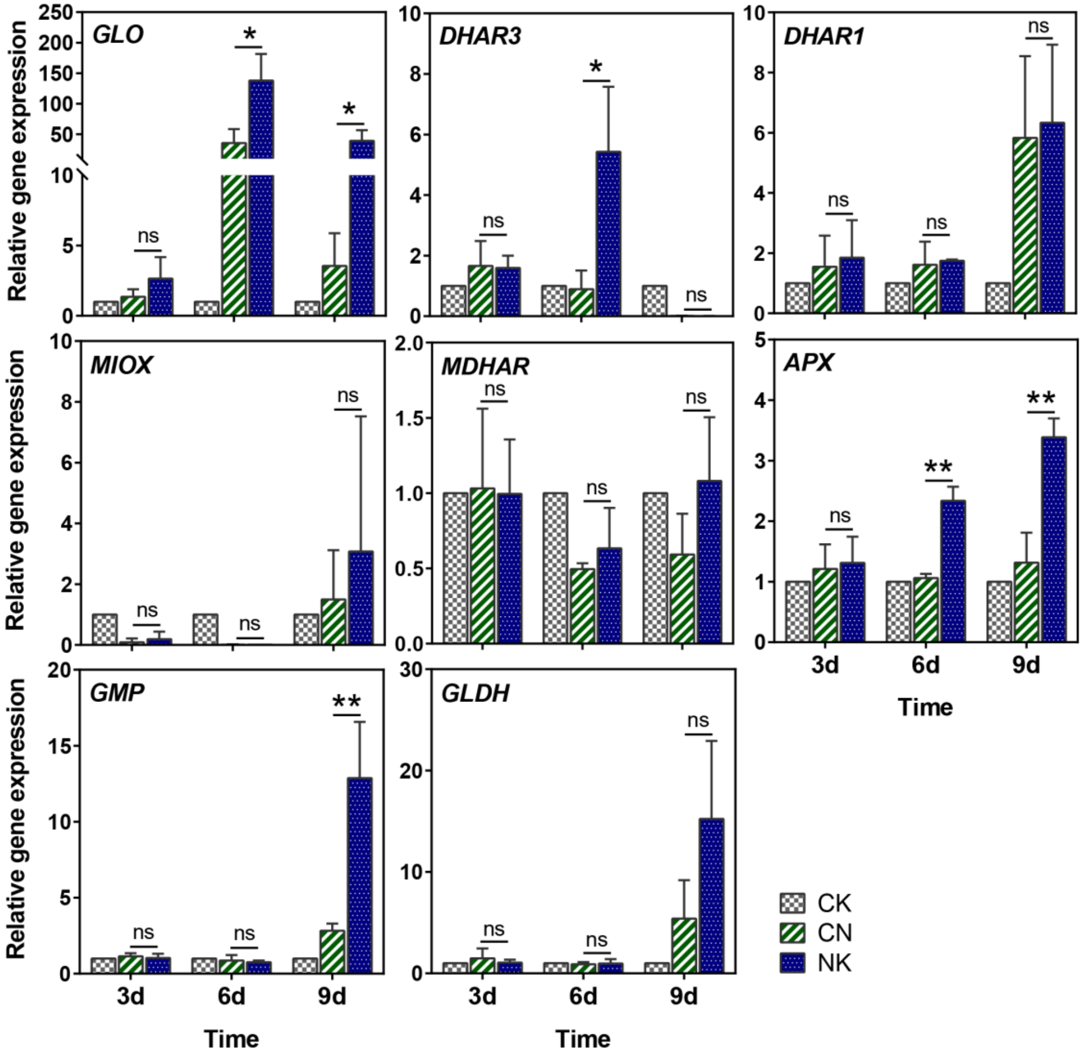


**Supplementary Figure 3.** Gene expression analysis. Each datum was normalized against the respective datum of the CK. $Na^+$, seedlings were cultivated in 1/2 Hoagland nutrient solution supplemented with NaCl (100 mM). $Na^+$+2KGA, 1/2 Hoagland nutrient solution was supplemented with NaCl (100 mM) and 2KGA (1 mM). *GLO*, *L-gulono-1,4-lactone oxidase*; *DHAR3*, *dehydroascorbate reductase-3*; *DHAR1*, *dehydroascorbate reductase-1*; *MIOX*, *Myo-inositol oxygenase*; *MDHAR*, *monodehydroascorbate reductase*; *APX*, *ascorbate peroxidase*; *GMP*, *GDP-mannose pyrophosphorylase*; *GLDH*, *L-galactose-1,4-lactone dehydrogenase*; ns, not significant, * $p < 0.05$, ** $p < 0.01$.

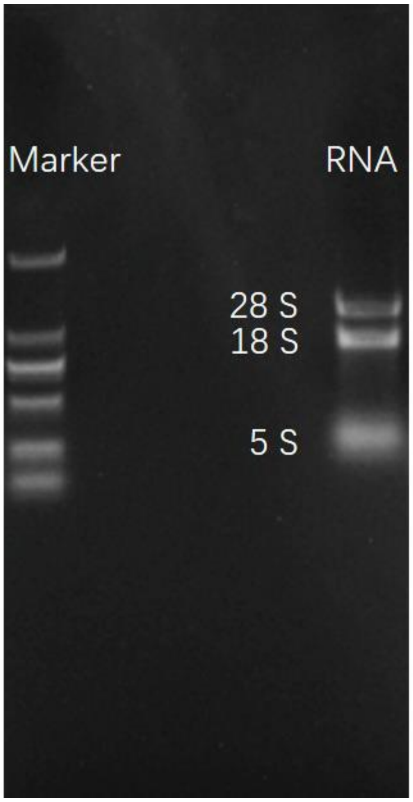


**Supplementary Figure 4.** Agarose gel electrophoresis of RNA.

## Supplementary Tables

**Supplementary Table 1.** Primers sequences

| Primer | Sequence (5' - 3') |
|---|---|
| APX-F | TCTTCAGGACACTGCTCAGG |
| APX-R | AACGCTGGTCTCGTTAATGC |
| DHAR1-F | AGGGAAAGTGCCAGTGCTTA |
| DHAR1-R | TGAGTGACGGCTCAGGATAC |
| DHAR3-F | TTGAGTGACGAGGTCCAACA |
| DHAR3-R | GCGCAAACAGAGGATGTGAT |
| GLDH-F | TCCGGCTGGTTAAAGTTCCT |
| GLDH-R | GCCAAACACAAGAAGGCTCA |

| | |
|---|---|
| GLO-F | CATAGAAGGGAGCGAGCAGA |
| GLO-R | TCCATTGATGTCCACAGCCT |
| GMP-F | TCCAGGGTGTGTTGTTGAGT |
| GMP-R | CAATCCTAGCCCACTGTCCA |
| MDHAR-F | CCATCTCAAAGGCGACACAG |
| MDHAR-R | ACTCACCACTGTCTCAGCAA |
| MIOX-F | GAATATTGGCCGGGTCGAAG |
| MIOX-R | CCTTCTCCAAACCGCTGAAG |
| Actin-F | TGTGCCAATCTACGAGGGTTT |
| Actin-R | TTTCCCGCTCTGCTGTTGT |

**Supplementary Table 2.** Absolute results of organic solutes, ASCAc, antioxidant enzymes, $H_2O_2$, and MDA.

| Time (d) | CK | | | $Na^+$ | | | $Na^+$+2KGA | | | |
|---|---|---|---|---|---|---|---|---|---|---|
| 3d | 8.4 | 9.8 | 8.9 | 10.5 | 11.5 | 11.0 | 11.9 | 11.7 | 11.7 | SP (mg/g FW) |
| 6d | 9.0 | 10.1 | 9.6 | 11.0 | 10.8 | 11.1 | 11.8 | 12.2 | 12.0 | |
| 9d | 9.2 | 10.0 | 9.6 | 12.4 | 12.8 | 12.6 | 12.9 | 13.5 | 13.2 | |
| 3d | 6.9 | 6.5 | 6.7 | 8.7 | 9.2 | 8.4 | 8.5 | 8.3 | 8.9 | SC (mg/g FW) |
| 6d | 4.4 | 4.6 | 4.5 | 4.5 | 4.2 | 4.5 | 8.1 | 8.3 | 7.4 | |
| 9d | 4.5 | 4.5 | 4.8 | 3.6 | 3.8 | 4.1 | 7.3 | 7.5 | 8.0 | |
| 3d | 7.2 | 5.7 | 7.3 | 13.6 | 12.1 | 11.1 | 11.2 | 11.7 | 13.2 | Proline (μg/g FW) |
| 6d | 17.4 | 14.8 | 15.9 | 100.2 | 97.5 | 99.1 | 102.3 | 106.6 | 100.9 | |
| 9d | 17.1 | 19.8 | 21.2 | 72.1 | 81.1 | 81.4 | 78.9 | 84.5 | 80.8 | |

| | | | | | | | | | | |
|---|---|---|---|---|---|---|---|---|---|---|
| 3d | 484.3 | 493.7 | 469.7 | 500.2 | 488.3 | 509.6 | 575.7 | 600.4 | 565.1 | |
| 6d | 357.3 | 377.7 | 349.7 | 327.5 | 317.2 | 345.4 | 360.2 | 349.3 | 364.5 | ASCAc (μg/g FW) |
| 9d | 379.2 | 372.1 | 389.1 | 324.9 | 315.1 | 337.2 | 335.9 | 345.8 | 335.1 | |
| 3d | 307.6 | 275.9 | 291.3 | 313.7 | 311.9 | 298.1 | 309.7 | 332.7 | 325.8 | |
| 6d | 358.7 | 314.6 | 306.1 | 289.6 | 298.4 | 295.3 | 300.0 | 322.0 | 316.1 | SODa (U/g FW) |
| 9d | 347.8 | 357.5 | 372.1 | 371.4 | 349.5 | 377.4 | 384.5 | 404.6 | 395.9 | |
| 3d | 522.6 | 618.4 | 540.0 | 588.0 | 659.0 | 622.5 | 677.4 | 695.1 | 677.0 | |
| 6d | 470.3 | 542.5 | 545.8 | 604.6 | 641.4 | 613.4 | 726.6 | 647.7 | 674.3 | PODa (U/g FW) |
| 9d | 616.3 | 562.5 | 602.8 | 584.5 | 714.5 | 608.6 | 751.5 | 771.1 | 731.3 | |
| 3d | 3.6 | 3.3 | 3.3 | 5.8 | 6.9 | 6.6 | 7.8 | 8.6 | 8.2 | |
| 6d | 16.2 | 17.5 | 15.5 | 16.7 | 17.9 | 17.0 | 18.4 | 19.2 | 19.8 | APXa (U/g FW) |
| 9d | 14.6 | 15.1 | 16.3 | 21.3 | 19.2 | 20.0 | 20.0 | 22.4 | 24.2 | |
| 3d | 10.5 | 11.2 | 8.9 | 13.0 | 11.5 | 12.4 | 16.6 | 17.1 | 15.0 | |
| 6d | 7.6 | 7.0 | 7.8 | 13.7 | 14.3 | 12.7 | 17.7 | 14.7 | 17.3 | CATa (U/g FW) |
| 9d | 14.7 | 14.4 | 11.6 | 13.7 | 18.5 | 17.1 | 21.1 | 20.3 | 19.2 | |
| 3d | 4.6 | 4.4 | 4.5 | 5.3 | 5.5 | 5.5 | 5.7 | 5.9 | 5.5 | |
| 6d | 4.7 | 4.9 | 4.9 | 6.1 | 6.0 | 6.2 | 5.7 | 5.9 | 5.7 | $H_2O_2$ (μg/g FW) |
| 9d | 4.6 | 4.5 | 4.2 | 5.5 | 5.6 | 5.7 | 4.8 | 4.5 | 4.8 | |
| 3d | 4.3 | 4.4 | 4.5 | 6.1 | 7.2 | 6.4 | 6.6 | 6.9 | 6.6 | |
| 6d | 4.6 | 4.2 | 4.2 | 6.2 | 6.6 | 6.4 | 6.1 | 6.2 | 5.9 | MDA (μmol/g FW) |
| 9d | 3.9 | 4.1 | 4.0 | 6.1 | 6.2 | 6.0 | 5.2 | 5.7 | 5.8 | |

SC, soluble carbohydrate; SP, soluble protein; ASCAc, L-ascorbic acid; SODa, superoxide dismutase activity; PODa, peroxidase activity; APXa, ascorbate peroxidase activity; CATa, catalase activity; $H_2O_2$, hydrogen peroxide; MDA, malondialdehyde; FW, fresh weight.

**Supplementary Table 3.** The purity of RNA and cDNA.

| | RNA | | cDNA | |
|---|---|---|---|---|
| | 260/280 | 260/230 | 260/280 | 260/230 |
| 1 | 1.960 | 1.997 | 1.771 | 2.129 |
| 2 | 1.942 | 1.917 | 1.770 | 2.126 |
| 3 | 1.997 | 2.023 | 1.773 | 2.128 |
| 4 | 1.996 | 2.014 | 1.772 | 2.126 |
| 5 | 1.920 | 1.950 | 1.763 | 2.086 |
| 6 | 1.946 | 2.005 | 1.773 | 2.125 |
| 7 | 1.922 | 1.916 | 1.776 | 2.122 |
| 8 | 1.991 | 1.982 | 1.772 | 2.115 |
| 9 | 1.968 | 1.924 | 1.774 | 2.120 |
| 10 | 1.927 | 1.970 | 1.773 | 2.117 |
| 11 | 1.949 | 2.005 | 1.773 | 2.114 |
| 12 | 1.960 | 1.969 | 1.773 | 2.114 |
| 13 | 1.968 | 1.996 | 1.777 | 2.124 |
| 14 | 2.025 | 1.966 | 1.769 | 2.118 |
| 15 | 2.000 | 2.006 | 1.773 | 2.120 |
| 16 | 1.958 | 1.968 | 1.774 | 2.111 |
| 17 | 1.959 | 1.996 | 1.778 | 2.122 |

| | | | | |
|---|---|---|---|---|
| 18 | 1.993 | 2.012 | 1.773 | 2.118 |
| 19 | 2.082 | 1.984 | 1.770 | 2.125 |
| 20 | 2.025 | 1.959 | 1.777 | 2.125 |
| 21 | 2.053 | 1.973 | 1.774 | 2.125 |
| 22 | 2.051 | 1.958 | 1.773 | 2.123 |
| 23 | 2.070 | 1.947 | 1.766 | 2.115 |
| 24 | 2.021 | 2.008 | 1.774 | 2.121 |
| 25 | 1.971 | 2.116 | 1.767 | 2.066 |
| 26 | 1.968 | 2.083 | 1.765 | 2.091 |
| 27 | 1.971 | 2.117 | 1.775 | 2.044 |

## Supplementary Methods

### Supplementary Method 1

### Analysis of photosynthetic pigments

Leaf samples (0.5 g) were ground in the mortar with a small amount of quartz sand, calcium carbonate and 1 mL ethanol. After being ground evenly, 4 mL ethanol was added to the mortar to prepare homogenate. The homogenate was centrifuged at 4 °C (7,200 × g) for 5 min. Then, the supernatant was transferred to 10 mL volumetric flask, the precipitate was suspended with 2 mL ethanol and centrifuged again. The supernatant obtained again was also transferred to the volumetric flask. Finally, ethanol was added to the volumetric flask to 10 mL as the extract. The absorbance of the extract was measured at 470, 649, and 665 nm, respectively. Ca, Cb, Cab and Ccar represented Chlorophyll a (Chla), chlorophyll b (Chlb), total chlorophyll (Chlab), and carotenoid (Car) contents. The calculation formulas were as follows:

Ca = 13.36 × $A_{665}$ - 5.19 × $A_{649}$ (μg/mL)

Cb = 27.43 × $A_{649}$ - 8.12 × $A_{665}$ (μg/mL)

Cab = 5.24 × $A_{665}$ + 22.24 × $A_{649}$ (μg/mL)

Ccar = (1000 × $A_{470}$ - 2.13 × Ca - 97.64 × Cb) / 209 (μg/mL)

### Supplementary Method 2

**Method for determining soluble protein (SP)**

Assay method of SP: Leaf samples (0.5 g) were ground uniformly with liquid nitrogen and then 5 mL desalting water was added to obtain homogenate. The homogenate was centrifuged at room temperature (7,200 × *g*) for 5 min, and the supernatant was taken as the extract. 1 mL extract was mixed with 5 mL Coomassie Brilliant Blue solution (100 mg Coomassie Brilliant Blue solution was dissolved in a small amount of 90% ethanol, and 100 mL phosphoric acid was added and then desalinated water was added to 1 L) and let stood at room temperature for 2 min. Then, the absorbance was measured at 595 nm. The SP content was calculated according to the standard curve of bovine serum protein.

**Supplementary Method 3**

**Method for determining proline.**

Assay method of proline: leaf samples (0.5 g) were placed into a 10 mL centrifuge tube and 5 mL sulfosalicylic acid solution was added. Then the proline was extracted in boiling water bath for 10 min, and cooled to room temperature. 2 mL extract was transferred into a 15 mL centrifuge tube, and 2 mL glacial acetic acid and 2 mL acidic ninhydrin solution (1.25 g ninhydrin + 30 mL glacial acetic acid + 20 mL 6 mol/L phosphoric acid) were added. The mixture was bathed in boiling water for 20 min, then cooled to room temperature and added with 4 ml toluene. After shaking for 1 min, it was centrifuged (7,200 × *g*) for 5 min to take the supernatant as the extract. The absorbance was measured at 520 nm, and the content of proline in samples was calculated by a proline standard curve.

**Supplementary Method 4**

**Antioxidant enzyme activity**

The reaction system of SODa determination was as follows: 50 μL crude enzyme solution + 2 mL 39 mM methionine (methionine was dissolved in PBS buffer A) + 2 mL 0.225 mM p-Nitro-Blue tetrazolium chloride (NBT) aqueous solution + 1 mL 0.6 mM EDTA-Na + 1 mL 0.012 mM riboflavin. The absorbance was measured at 560 nm after 20 min of illumination. Enzyme activity was defined as 50% inhibition of NBT photoreaction as one enzyme activity unit (U). The calculation formula was as follows:

$$A = ((D1 - D2) \times V \times 1000 \times 60) / (D1 \times B \times W \times T \times 50\%)$$

A, enzyme activity; D1, the absorbance measured when the enzyme was inactivated; D2, the absorbance was measured when the enzyme was active; V, total volume of crude enzyme solution; 1000 = 1000 μL; 60 = 60 min; B, dose of crude enzyme solution; W, weight of sample; T, time of reaction.

The reaction system of APXa determination was as follows: 285 μL PBS buffer A + 5 μL 4 mM $H_2O_2$ + 10 μL crude enzyme solution. The absorbance value was recorded at 290 nm for 30 consecutive times with an interval of 10 seconds. Enzyme activity was defined as a 0.001 change in absorbance per second as one enzyme activity unit (U).

The reaction system of CATa determination was as follows: 285 μL PBS buffer A + 5 μL 1.5 mM $H_2O_2$ + 10 μL crude enzyme solution. The absorbance value was recorded at 240 nm for 7 consecutive times with an interval of 1 min. Enzyme activity was defined as a 0.01 change in absorbance per minute as one enzyme activity unit (U).

The reaction system of PODa determination was as follows: 1 mL crude enzyme solution + 3 mL 20 mM pH 7.2 PBS buffer B (500 mL PBS buffer A + 0.28 mL guaiacol + 0.19 mL $H_2O_2$). The absorbance value was measured at 465 nm. Recorded the change of absorbance within 5 minutes. Enzyme activity was defined as a 0.01 change in absorbance per minute as one enzyme activity unit (U).
